# Supplementary material for: Effects of Mild Excitotoxic Stimulus on Mitochondria Ca2+ Handling in Hippocampal Cultures of a Mouse Model of Alzheimer’s Disease
Source: Cells. 2021 Aug 10;10(8):2046. doi: 10.3390/cells10082046 (PMC8394681; doi:10.3390/cells10082046)
Supplement: Supplementary file 1 [file cells-10-02046-s001.zip › cells-1305684-supplementary.pdf]

Supplementary figures

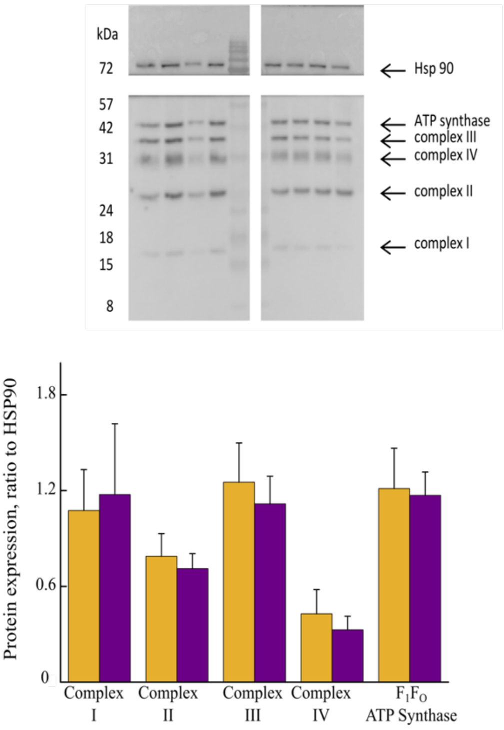

S1 Protein expression and quantification of ETC and F_1F_OATP-synthase components

Panel A Western blot of protein extracts derived from 4 independent cultures for WT (left) and B6.152H (right), probed with antibodies recognizing specific components of the electron transfer chain, and F_1F_O ATP-synthase. Antibody against HSP 90 was used as loading control.

Panel B: quantification of protein expression for each component: results expressed as mean± SD. Mann-Whitney non-parametric test: differences not statistically significant within each pair.

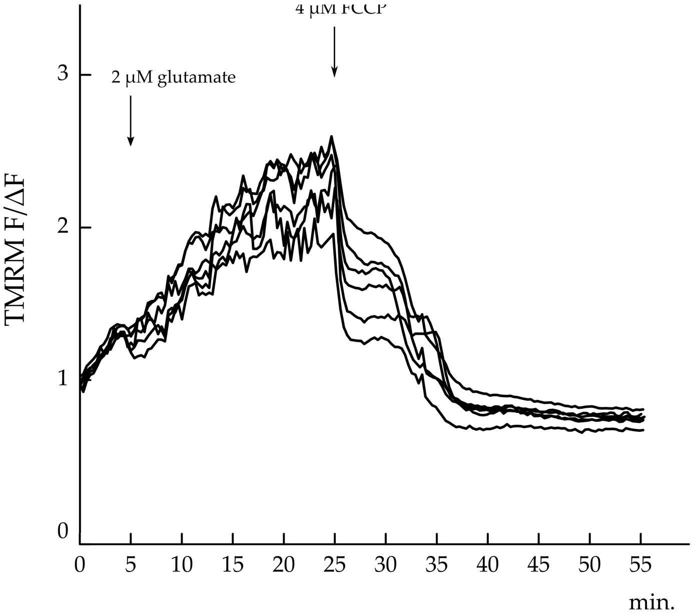

S2 Effect of glutamate treatment on WT neuronal culture in the presence of Alisporivir

Representative traces of WT cells treated with 1.5 μM Alisporivir, loaded with the potentiometric probe TMRM. Were indicated 2 μM glutamate and 4 μM FCCP were added. Time point TMRM acquisitions are normalized to the difference between the average of initial fluorescence (first 5 minutes acquisitions), minus the average of fluorescence signal measured after the addition of the uncoupler FCCP (last 3 minutes acquisitions).
